# Hemizygous *FLNA* variant in West syndrome without periventricular nodular heterotopia

**DOI:** 10.1038/s41439-020-00131-9

**Published:** 2020-12-03

**Authors:** Yoshitaka Hiromoto, Yoshiteru Azuma, Yuichi Suzuki, Megumi Hoshina, Yuri Uchiyama, Satomi Mitsuhashi, Satoko Miyatake, Takeshi Mizuguchi, Atsushi Takata, Noriko Miyake, Mitsuhiro Kato, Naomichi Matsumoto

**Affiliations:** 1 Department of Human Genetics, Yokohama City University Graduate School of Medicine, 3-9 Fukuura, Kanazawa, Yokohama, 236-0004 Japan; 2 Department of Pediatrics, Fukushima Medical University School of Medicine, Fukushima, 960-1295 Japan; 3 Department of Pediatrics, Ohara General Hospital, Fukushima, 960-8611 Japan; 4 Department of Oncology, Yokohama City University Graduate School of Medicine, Yokohama, 236-0004 Japan; 5 Department of Pediatrics, Showa University School of Medicine, Tokyo, 142-8555 Japan

**Keywords:** Genetics, Neurological disorders

## Abstract

Pathogenic *FLNA* variants can be identified in patients with seizures accompanied by periventricular nodular heterotopia (PVNH). It is unusual to find *FLNA* aberrations in epileptic patients without PVNH on brain imaging. We report a boy with cryptogenic West syndrome followed by refractory seizures and psychomotor delay. We performed whole-exome sequencing and identified a de novo missense variant in *FLNA*. It is noteworthy that this patient showed no PVNH. As no other pathogenic variants were found in epilepsy-related genes, this *FLNA* variant likely caused West syndrome but with no PVNH.

In epileptic encephalopathy, epileptic activity contributes to severe cognitive and behavioral impairments^1^. Genetic causes can be detected in patients with epileptic encephalopathy, including age-dependent epilepsy in infancy such as West syndrome^2^.

The *FLNA* gene at Xq28 encodes the filamin A protein, which is known to interact with more than 90 other proteins that could involve neuronal migration and other functions^3–5^.

Pathogenic *FLNA* variants are known to cause several human phenotypes^6^. Loss-of-function variants in *FLNA* cause periventricular nodular heterotopia (PVNH1) (MIM #300017) or/and congenital intestinal pseudo-obstruction. Gain-of-function mutations in *FLNA* cause otopalatodigital spectrum disorders. Cardiac valvular dystrophy is observed in patients with both loss-of-function and gain-of-function variants, suggesting a different mechanism involved in valvular dystrophy. *FLNA* pathogenic variants could also cause thrombocytopenia through aberrant activation of GPIbα and αIIbβ3 integrin, which are receptors of FLNa and essential to platelet adhesion and aggregation^4^.

Seizure is a common symptom in PVNH patients (more than 70%)^7^. Based on the X-linked dominant inheritance pattern of *FLNA* aberration, *FLNA*-related PVNH patients are usually females (more than 90%)^7^, and *FLNA* variants in males might be lethal in association with high miscarriage rates in mothers affected with PVNH1 as well as high infantile mortality in affected boys^8,9^. The reason for such high mortality remains unclear, but early deaths in males could arise from hemorrhage^3^ or cardiovascular malformation^10^ but not from brain malformation. However, approximately 30 male patients whose *FLNA* variants are predicted to be partially loss-of-function or mosaic have been reported to date^11^. The Flna-null mouse model showed that they died at midgestation with widespread hemorrhage from abnormal vessels and truncus arteriosus^12^.

Here, we report a boy with West syndrome arising from a de novo *FLNA* variant detected by whole-exome sequencing (WES), but no PVNH was seen by brain MRI.

All human studies were approved by the institutional review boards of Yokohama City University, Showa University and Fukushima Medical University. Written informed consent was obtained from the parents of the patient.

WES was performed on the patient’s DNA. Genomic DNA was captured by the SureSelect Human All Exon v6 system (Agilent Technologies, Santa Clara, CA, USA) and sequenced on the HiSeq 2500 platform (Illumina, San Diego, CA, USA) as described previously^13^. The mean WES coverage was 71.07x, and at least 89.8% coverage of the target regions with 20 or more reads was achieved. To identify causative variants of infantile spasms, we narrowed down variants in our patient based on the allele frequency (<0.001 for autosomal dominant model, <0.01 for autosomal recessive model, and <0.01 for X-linked model) using the Human Genetic Variation Database (HGVD) (http://www.hgvd.genome.med.kyoto-u.ac.jp/), the Exome Aggregation Consortium (ExAC) (http://exac.broadinstitute.org/), the Tohoku Medical Megabank Organization (ToMMo) (https://www.megabank.tohoku.ac.jp/english/) and the Genome Aggregation Database (gnomAD) (https://gnomad.broadinstitute.org/). We also used our in-house whole-exome database of 575 Japanese control individuals and excluded nonpathogenic variants by their allele frequency. After selecting the variants in the database according to frequency, we determined whether the remaining variants were deleterious using three prediction tools recommended in the American College of Medical Genetics and Genomics (ACMG) Standards and Guidelines: SIFT (https://sift.bii.a-star.edu.sg/), Polyphen-2 (http://genetics.bwh.harvard.edu/pph2/) and CADD (https://cadd.gs.washington.edu/).

Copy number variations (CNVs) were also detected from the WES data using the eXome-Hidden Markov Model as previously described^14^.

To determine whether the deleterious variant detected by WES was a de novo variant, we sequenced the DNA of the parents and patient using the Sanger method. For polymerase chain reaction (PCR) analysis, we used Takara Ex Taq HS polymerase (Takara Bio, Shiga, Japan) and two primers (forward 5′-CTT TTG GGC CAT AGC AGT TAA GA-3′; reverse 5′-CAG TGC ACT TGC TGG CGT CC-3′) with the following PCR conditions: denaturation at 94 °C for 30 s; annealing at 68 °C for 30 s; and extension at 72 °C for 30 s for 35 cycles.

The paternal and maternal DNA were examined by fragment analysis in twelve different regions that included STRs as previously described^15^.

A 3-year-old boy was born to nonconsanguineous parents with no family history of seizures or other neurological disorders. His full-term birth height and weight were 49.5 cm and 3.225 g, respectively, which are within ±2.0 SD. The patient had an uneventful perinatal period. He had transient bilateral dystonic posture in the upper limbs at the age of 5 months. At 9 months, he developed infantile spasms. Hypsarrhythmia and spasms were observed on electroencephalography (EEG) (Fig. 1a, b). Thoracoabdominal X-ray photograph, echocardiogram, fundus examination and biochemical examination showed no abnormalities. Administration of adrenocorticotropic hormone (ACTH) improved the abnormal waveforms on interictal EEG (Fig. 1c) but could not control spasms. After ACTH therapy, vitamin B6, zonisamide, valproic acid, clonazepam, topiramate, vigabatrin, and lamotrigine were administered but were not effective against his spasms. Furthermore, a corpus callosotomy at the age of 21 months had no effect on his spasms. PVNH was not observed on brain MRI either before or after corpus callosotomy (Fig. 1d–k). The patient had a developmental delay (DQ = 28) and could not speak any meaningful words. He also exhibited autistic behavior. Skeletal abnormalities were not observed.

**Fig. 1:**
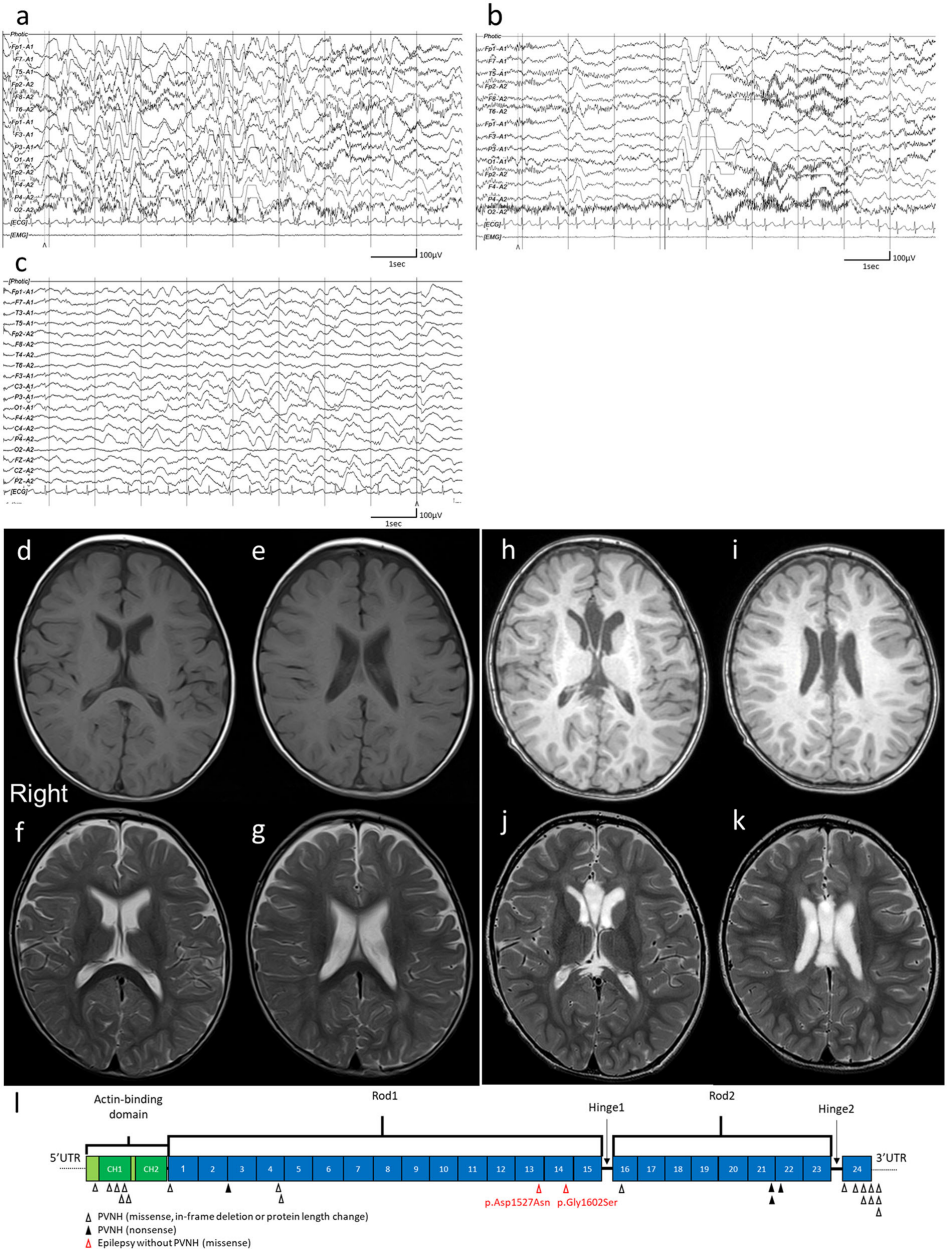
Clinical information of the patient and structure of filamin A protein. **a** Electroencephalography (EEG) results of the patient showing hypsarrhythmia during wakefulness at 9 months of age. **b** Ictal EEG results showing cluster spasms with head drop at 9 months of age. **c** EEG results at 11 months of age and after adrenocorticotropic hormone therapy. Sporadic slow waves were observed in the bitemporal regions. **d**, **e** Brain MRI T1-weighted and **f**, **g** T2-weighted images of the patient at the age of 15 months. Three pediatric neurologists independently confirmed the absence of periventricular nodular heterotopia. **h**, **i** Brain MRI T1-weighted and **j**, **k** T2-weighted images of the patient at the age of 29 months. **l** Filamin A protein with functional domains and FLNA variants found in males. Functional domains consist of the actin-binding domain containing two calponin homology (CH) domains and 24 Ig domains. Pathogenic variants detected in males are shown as triangles below Filamin A. FLNA variants in PVNH1 or in epilepsy without PVNH are shown as black or red triangles. Filled or open triangles indicate nonsense or missense/in-flame changes.

WES showed a rare hemizygous missense variant in *FLNA* (NM_001456.4: c.4804G>A: p.Gly1602Ser) (Fig. S1a, b). Sanger sequencing of the patient’s parents confirmed that the variant was de novo. Additionally, short tandem repeat analysis indicated that they were his biological parents.

This variant was not registered in the in-house database, HGVD, ExAC, ToMMo or gnomAD and was indicated as pathogenic based on the following in silico tools: SIFT: 0 (damaging), Polyphen-2: 0.996 (deleterious) and CADD: 27.0 (deleterious). No other rare variants in epilepsy-related genes registered in OMIM or deleterious CNVs were found in the patient. In addition, amino acid substitutions are highly evolutionarily conserved among different species (Fig. S1c).

On the basis of the ACMG Standards and Guidelines^16^, we concluded that this variant is likely pathogenic according to the following evidence of pathogenicity: strong: PS2, moderate: PM2, supporting: PP3.

Among the clinical consequences of *FLNA* variants, PVNH1 is the most common brain abnormality^6^. However, we could not detect PVNH or other brain MRI abnormalities in this patient who developed infantile spasms. We found at least three cases of epilepsy arising from possibly pathogenic *FLNA* variants that provided no description of PVNH or other abnormal MRI findings (but with no images presented) in the literature^17–19^ (Table 1). One reported male case had a p.Asp1527Asn variant within the Ig domain^18^ and our case variant, p.Gly1602Ser, was also located within the neighboring Ig domain (Fig. 1l). Two independent male cases could support that *FLNA* variants can cause epileptic encephalopathy with no PVNH. Interestingly, seizures associated with PVNH1 patients (females and males) are typically adolescent-onset, and early infantile onset is uncommon^6,7,10,11,20^. Our patient started spasms at the age of 9 months, and other epileptic patients without PVNH had seizures before the age of 1 year (Table 1).

**Table 1:** Epileptic Individuals with no periventricular nodular heterotopia arising from *FLNA* variants.

	Case 1	Case 2^a^	Case 3	Our Case
Age at onset	9 months	5 months	Unknown	9 months
Sex	Female	Male	Female	Male
Mutation	c.5324C>T p.Leu1775Pro de novo	c.4579G>A p.Asp1527Asn maternal inherited	c.2662G>T p.Glu888* heterozygous	c.4804G>A p.Gly1602Ser^b^ de novo hemizygous
Phenotype	Lennox-Gastaut syndrome	Epileptic encephalopathies, infantile	Epilepsy (generalized or focal)	Cryptogenic West syndrome
Allele frequency (number of homozygotes, hemizygotes) in gnomAD	0 (0,0)	8.83e−5 (0,2)	0 (0,0)	0 (0,0)
SIFT	0.22	0.21	-	0
Polyphen2	0.905	0.452	-	0.996
CADD	22.9	23.4	41	27.0
Brain imaging	Parietal venous angioma	Normal	Not available	Normal
Source	Allen et al.^17^	Wei et al.^18^	DiFrancesco et al.^19^	This report

^a^Case 2, from a paper written in Chinese, is a boy diagnosed with moderate infantile epileptic encephalopathy with an onset age of 5 months. Wei et al. created a customized kit covering all exonic regions associated with 4000 monogenic genetic diseases in the OMIM databases, performed NGS using the Illumina platform, and detected an *FLNA* heterozygous missense variant. They described that the *FLNA* variant is likely pathogenic in association with PVNH1, but no abnormality was seen in brain MRI.

^b^In gnomAD, there is a missense variant (allele frequency: 1.11e−5 and number of hemizygotes: 1) that affects the same amino acid but leads to a different amino acid substitution (p.Gly1602Arg). In silico scores of that variant are SIFT: 0, Polyphen: 0.999 and CADD: 28.1.

While PVNH1 patients often suffer from seizures, it is unclear whether heterotopia is a direct cause of these seizures. The extent of PVNH on brain MRI is not associated with the age of onset of seizures or overall clinical severity as previously described^7^. *Flna* transcripts are highly expressed across the entire cerebral cortex in the late period of mouse embryogenesis (E14.5–E16.5), while filamin B, a homolog of filamin A, is localized near the ventricular and subventricular zone^21^. Interestingly, in the late period of embryogenesis (E14.5) of *Flna*-null mice, no neuronal accumulation in the ventricular zone or heterotopic neurons was recognized^12^. Filamin A is known to interact with the HCN1 channel and modulate neuronal excitability in the mature brain via endocytosis of the HCN1 channel^22^ encoded by *HCN1*. *HCN1* is also expressed in the entire brain (especially in the cerebral cortex, hippocampus and cerebellum) of rats^23^. *HCN1* variants cause early infantile epileptic encephalopathy (EIEE24, #615871). Considering these facts, *FLNA* abnormalities may cause subventricular zone abnormalities leading to PVNH1 and dysfunction of the entire cerebral cortex.

We report a boy who suffered from West syndrome without PVNH1 arising from a de novo missense variant in *FLNA*. Considering the wide expression of filamin A protein in the mature brain, *FLNA1* variants may be considered one of rare causes of epileptic encephalopathy without PVNH1.

## Supplementary information

Figure S1

## Data Availability

The relevant data from this Data Report are hosted at the Human Genome Variation Database at 10.6084/m9.figshare.hgv.2945.
