# Supplementary material for: Hemizygous FLNA variant in West syndrome without periventricular nodular heterotopia
Source: Hum Genome Var. 2020 Dec 3;7:43. doi: 10.1038/s41439-020-00131-9 (PMC7713383; doi:10.1038/s41439-020-00131-9)
Supplement: Supplementary file 1 — Figure S1 [file 41439_2020_131_MOESM1_ESM.pdf]

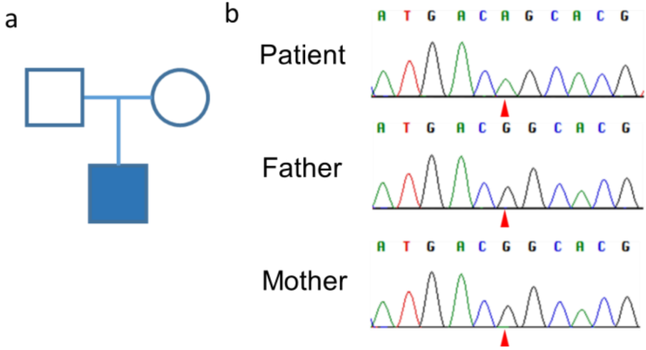

c

PKKTHIQDNHD**G**TYTVAYVPDVT 1602 Human
PKKTHIQDNHD**S**TYTVAYVPDVT 1602 mutated
PKKTHIQDNHD**G**TYTVAYVPDVT 1602 Mmulatta
PKKTHIQDNQD**G**TYTVAYVPDVT 1313 Fcatus
PKKTHIQDNHD**G**TYTVAYVPDVP 1602 Mmucculus
PKKANIRDNQD**G**TYLVSYVPDMT 1573 Drerio
PKKATIRDNQD**G**TYTVSYVPDMT 1602 Xtropicalis
